# Rod and cone interactions in the retina

**DOI:** 10.12688/f1000research.14412.1

**Published:** 2018-05-23

**Authors:** Gordon Fain, Alapakkam P. Sampath

**Affiliations:** 1 Department of Ophthalmology, Jules Stein Eye Institute, David Geffen School of Medicine, University of California Los Angeles, 100 Stein Plaza, Los Angeles, CA 90095-7000, USA; 2 Department of Integrative Biology and Physiology, University of California Los Angeles, Terasaki Life Sciences, 610 Charles E. Young Drive South, Los Angeles, CA 90095-7239, USA

**Keywords:** retina, photoreceptor, processing, interaction

## Abstract

We have long known that rod and cone signals interact within the retina and can even contribute to color vision, but the extent of these influences has remained unclear. New results with more powerful methods of RNA expression profiling, specific cell labeling, and single-cell recording have provided greater clarity and are showing that rod and cone signals can mix at virtually every level of signal processing. These interactions influence the integration of retinal signals and make an important contribution to visual perception.

When one of us was a graduate student in the early ’70s, Edwin Land, the inventor of the Polaroid camera, came to Harvard to give a seminar on his “retinex” theory of color vision ^1, 2^. Though a successful entrepreneur and businessman, Land retained an immense curiosity about the eye and especially about color. His retinex theory attempted to account for what most scientists would call color constancy—the remarkable ability of the visual system to maintain the perceived color of an object in quite different conditions of illumination. Apples continue to be red or green at dawn and at noon, even though the light reflected from the apples at different times of day can be quite different in wavelength composition.

After the seminar, Gordon told Land about his experiments on interactions between rod and cone signals in the mudpuppy retina ^3^, and Land invited him to the research laboratory of the Polaroid Corporation to meet Land’s associate John McCann, who was working on rod contributions to color vision. Although we are accustomed to think of rods as being responsible for dim-light vision and cones for color vision, McCann had devised a psychophysical demonstration with light stimulating only the rods and long-wavelength cones. He showed with little doubt that rods could contribute to the perception of hue ^4, 5^. His results and those of other psychophysicists (see, for example, ^6, 7^) have provided clear evidence of a contribution of rods to color.

In addition to these experiments, a large body of psychophysical literature indicates that signals derived from rods and cones can interact at many levels in the retina (see 8). Rod signals can influence cone spatial acuity and temporal sensitivity in sometimes quite surprising ways. Because rod signals are slower than cone signals, the signals from the two kinds of photoreceptors to flickering illumination can arrive at downstream targets out of phase with one another so that the flickering light stimulating both kinds of photoreceptors can seem to be steady ^9^. Even when the rods are not stimulated, they can decrease cone sensitivity to flicker during dark adaptation ^10^, and cones can depress rod sensitivity and move rod saturation to lower light intensities ^11^. These interactions probably occur somewhere in the retina, but nothing is known about the details of their mechanisms.

Renewed interest in interactions between rods and cones has been stimulated by several recent findings. Single-cell RNA expression profiling is being used in many parts of the nervous system to identify and distinguish different cell types from the profiles of the RNAs they express (see, for example, ^12, 13^). This method has also been used to good advantage on the retina, where it has now provided a comprehensive classification of all types of mouse bipolar cells ^14^. Careful examination of contacts of these cells with another technique—serial-section electron microscopy followed by computer-guided reconstruction of cell morphology ^15^—has given us a more complete understanding of rod and cone pathways through the mammalian retina and shown that they intermingle more than previously thought ^16^. Physiological studies have also provided new perspectives (for example, ^17^). Joesch and Meister ^18^ have shown, for example, that mice have a specific kind of ganglion cell (called the J-RGC or JAMB; see 19) with a color-opponent receptive field (see also 20). These cells resemble ganglion cells thought to mediate color vision in many vertebrates, but the center OFF response of the J-RGC can come specifically from ultraviolet-sensitive cones, and the ON surround from rods. The center and surround are mutually antagonistic, suggesting that the mouse retina contains specific microcircuits to provide color information resulting from interactions between rod and cone signals.

Another even more surprising finding is the claim of Tikidji-Hamburyan and colleagues ^21^ that rods can continue to function at much higher luminance than previously supposed. These investigators recorded from the retinas (and the central nervous system) of mice genetically engineered to lack cone function and showed that responses could be recorded from photoreceptors and ganglion cells even in bright, bleaching light. These findings challenge earlier psychophysical measurements in humans ( 22; see 23) and mice ^24^ as well as electrical recordings from single mammalian photoreceptors (for example, ^25– 27^) and from other retinal neurons ^18, 28^, all of which seem to show that rods saturate and become essentially non-functional in relatively dim background light to allow the cones with their kinetically faster responses to dominate perception.

To put this research into perspective and stimulate new avenues of research, we will endeavor to describe our present understanding of rod and cone interactions in the retina, emphasizing more recent findings from the mouse and primate. There are several demonstrated pathways for rod and cone signals to be communicated through the retina ( Figure 1), and each of these pathways provides opportunities for the two signals to influence one another.

**Figure 1.**
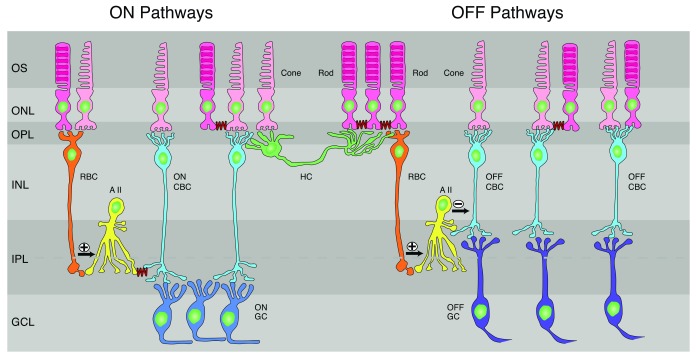
Rod and cone pathways through the mammalian (mouse) retina. **(Left)** ON pathways. Rod bipolar cell (RBC) receives input mostly from rods but also from cones and makes excitatory glutamatergic synapses onto AII amacrine cells, which in turn make gap junctions (squiggly lines) onto ON cone bipolar cells (ON CBCs). The ON CBCs then synapse onto ON-type ganglion cells (ON GCs). Rods also make gap junctions onto other rods and onto cones (squiggly lines), and the cones then carry rod signals to ON CBCs. **(Center)** ** Mouse horizontal cell (HC), whose dendrites contact exclusively cones and whose axon terminal contacts exclusively rods. Both cell body and axon can receive both rod and cone signals indirectly by passive spread through gap junctions between receptors. **(Right)** OFF pathways. RBCs make excitatory synapses onto AII amacrine cells, which make inhibitory glycinergic synapses onto OFF cone bipolar cells (OFF CBCs). These in turn synapse onto OFF ganglion cells (OFF GCs). Rods make gap-junctional contacts onto cones, which carry rod signals through OFF CBCs to OFF GCs. Finally, some OFF bipolar cells receive input from both rods and cones (far right). Dashed line through inner plexiform layer (IPL) indicates sublaminae; upper layer (sublamina a) contains terminations of OFF bipolar cells and dendrites of OFF GCs, and lower (sublamina b) contains ON bipolar cell terminals and ON GC dendrites ^42^. GCL, ganglion-cell layer; INL, inner nuclear layer; ONL, outer nuclear layer; OPL, outer plexiform layer; OS, outer segments of photoreceptors. After 43, 44.

## Electrical coupling of rods and cones

A first stage of interaction between rods and cones occurs within the photoreceptor layer itself. Rods are electrically coupled to cones in many vertebrate species ^29^, including teleost fish ^30^, amphibians ^31^, mice ^32, 33^, and primates ^34, 35^. Although the coupling is known to require connexin 36 ^36, 37^, and connexin 36 immunolabeling can be readily identified at gap junctions between rods and cones on the cone side of the junction ^33, 38, 39^, the identity of the rod connexin has long been uncertain. We think it quite likely, however, that the rod protein in the mouse is also some form of connexin 36. The evidence is, first, that connexin 36 expression has been shown to be widespread within the photoreceptor layer, and there is so much labeling that it is unlikely to be confined only within the cones ^36^. In addition, Jin and colleagues ^40^ have recently shown that coupling is essentially abolished when connexin 36 gene expression is deleted specifically within the rods. However, it remains possible that two different isoforms of connexin 36 are expressed in the two kinds of photoreceptors.

The preferred direction of current flow will be from rods to cones because of the much greater number of rods in most mammalian retinas ^41^. Therefore, the extent of coupling between rods and cones will set the magnitude of rod signals seen within the cone pedicle. Rod–cone coupling has some interesting features. Coupling of rods and cones in goldfish retina is under circadian control so that it is strong in darkness at night but much weaker in brighter illumination during the day ^30^. The extent of coupling is regulated by dopamine and D _2_ receptors, such that increases in dopamine and D _2_-receptor stimulation during the day decrease coupling. In primates, on the other hand, neither background light nor dopamine has been reported to influence the extent of coupling ^35^.

Asteriti and colleagues ^32^ have recorded from photoreceptors with perforated patch in mouse retina and observed that the amplitude of the rod voltage signal recorded in cones gradually increases over many minutes during the duration of the recording. The mechanism of this potential modulation is unclear. Our graduate student Norianne Ingram has recorded rod input in mouse cones with whole-cell patch recording ^45^ and has seen no evidence for time dependence of the rod input. Clearly, much more remains to be learned about the physiology of rod–cone coupling and its control by light intensity and circadian rhythm.

## Rod and cone inputs to bipolar cells

Rods and cones make synapses in the outer plexiform layer with two kinds of cells ( Figure 1): bipolar cells, whose axons transmit information to the next layer of retina called the inner plexiform layer, and horizontal cells, whose lateral processes interconnect photoreceptors and bipolar cells. Vertebrates have two kinds of bipolar cells (see 46): ON-type depolarizing to light in the center and hyperpolarizing to surrounding illumination and OFF-type hyperpolarizing to central illumination and depolarizing to the surround ^47, 48^. In teleost fish ^49^ and other lower vertebrates (for example, ^3, 50^), rods and cones synapse together onto the same ON and OFF bipolar cells, although some bipolar cells receive much more rod input than others.

In mammals, rod signals are conveyed down to the inner plexiform layer by only a few specific kinds of bipolar cells (see Figure 1 and 43). There is a single so-called “rod” bipolar cell, which is exclusively ON and which uses metabotropic mGluR6 synaptic receptors ^51^. Although these cells receive almost entirely rod input, we now know that they can also make a small number of contacts with cones ^16, 17^. These rod bipolar cells form the primary pathway of rod transit through the retina in dim light ^52– 54^ but can also continue to signal contrast modulation at ambient intensities above the cone threshold ^55^. It is currently unknown whether they can also respond in even brighter, bleaching light, when rods would be hyperpolarized well below their dark resting-membrane potential.

In addition to the rod bipolar, there are a further 13 or 14 kinds of bipolar cell in mouse ^14^, which can be either ON-type or OFF-type and use either mGluR6 glutamate receptors or ionotropic kainate/AMPA glutamate receptors. Most of these bipolar cells receive direct input exclusively from cones, but at least three types of OFF bipolar cell are also contacted by rods ^16, 56– 58^, in mouse as apparently also in primates ^59^. At present, there is little evidence for direct synaptic input of rods to ON bipolar cells other than the “rod” bipolar (but see 17). Each of the different bipolar cell types has a distinguishing response profile and different spatial and temporal characteristics ^60^. These differences are probably the combined result of differences in their populations of synaptic receptors, voltage-gated ion channels, receptor modulation, and inhibition from horizontal cells as well as from amacrine cells in the inner plexiform layer (see 46, 60, 61).

## Horizontal cells

Horizontal cells in lower vertebrates receive input from cones or from both rods and cones and can have light responses of two kinds. Some cells (called C-type) have mutually antagonistic input from two spectral classes of cones or from rods and cones, but most cells are L-type and receive exclusively hyperpolarizing input (see, for example, ^3, 62, 63^). Mammals seem not to have C-type horizontal cells but can have two morphologically different kinds of L-type horizontal cells: an A-type, which lacks an axon and contacts only cones, and a B-type, whose dendritic terminals contact only cones and whose often large axon terminal contacts only rods ^64, 65^. The mouse retina has only the B-type ( Figure 1; see 66).

Early recordings from mammalian horizontal cells nearly 50 years ago revealed that the same cell could receive both rod and cone input ^67^. Similar recordings from mice have also demonstrated that both the cell body and axon terminal of the B-type cell can receive input from rods and cones ^68^. Because the axon is slender and does not conduct action potentials, it is usually assumed that signals from the two kinds of photoreceptors cannot be conducted by the axon between the cell body and axon terminal ^69^ but must travel via gap junctions between the rod and cone photoreceptors before the photoreceptors synapse onto the horizontal cells. However, Trümpler and colleagues ^68^ have shown that cone signals can be recorded from horizontal-cell axon terminals in mice lacking connexin 36, suggesting that some signal can make it down the axon at least from the cell body to the axon terminal. They were unable to demonstrate that rod signals could be recorded from the cell bodies after elimination of the gap junctions. This result is surprising: if signal spreads from the cell body to the axon terminal, we should expect it to spread in the opposite direction as well, given the passive properties of signal conduction down the axon and the large size of the axon terminal relative to the cell body.

Horizontal cells provide inhibitory surrounds by feeding back onto both rods and cones (see, for example, ^70, 71^). Because both the soma and the axon terminals of horizontal cells receive signals from both kinds of photoreceptors, the rod signal in the horizontal-cell somata could feed back onto cones and the cone signal in the horizontal-cell axons could feed back onto rods. An effect of this kind was recently demonstrated by Szikra and colleagues ^28^, who showed that rod photoreceptors can be modestly depolarized by cone signals coming from horizontal cells. Because horizontal cells receive input from both kinds of photoreceptors, they are potentially capable of mediating antagonistic rod–cone interactions in either direction.

## Amacrine cells and the inner plexiform layer

Bipolar cells synapse onto both amacrine and ganglion cells in the next layer of processing, the inner plexiform layer ( Figure 1). There are at least 40 different types of amacrine cell, each with a distinguishable morphology and pattern of synaptic contact ^15^. Amacrine cells receive signals from rods or both rods and cones, either directly from bipolar cells or indirectly via the gap junctions between the two kinds of receptors. The A17 amacrine cells, for example, receive synapses almost exclusively from rod bipolar cells ^72^ and make reciprocal GABAergic inhibitory synapses back onto these same bipolar cells ^73, 74^. In this way, they provide local feedback inhibition at rod bipolar cell terminals ^75^, which can shape the temporal characteristics of the rod signal conveyed to the rest of the retina and visual system ^76^.

Rod and cone inputs have been particularly well studied for another amacrine cell, called the AII amacrine (or sometimes A2; see, for example, ^15^). In mammals, this cell serves an essential function in dim-light vision because rod bipolar cells do not make direct synaptic connection with ganglion cells. Instead, the rod bipolars make glutamatergic excitatory synapses onto AII amacrine cells, which then transfer the rod signal to cone ON bipolars via gap junctions (see Figure 1 and 46). The AII amacrine also makes glycinergic inhibitory synapses onto OFF cone bipolars (see also 54). As a result, the depolarizing signal of ON rod bipolars produces a depolarizing AII amacrine response, which then depolarizes ON cone bipolars and hyperpolarizes OFF cone bipolars. The AII amacrines also make gap junctions with one another, and, like the rod–cone junctions, these gap junctions are more conductive at night than during the day and are modulated by presynaptic activity ^77^ and by dopamine—but by D _1_ receptors instead of D _2_ receptors ^78, 79^.

This classic picture of rod signal flow is subject to several qualifications. First, AII amacrines also receive a relatively large direct synaptic input from OFF cone bipolar cells ^80– 82^. The functional consequence of this input remains largely unexplored. Second, the gap-junctional input from AII amacrines to ON cone bipolars can proceed in either direction ^83^; as a consequence, depolarization of ON cone bipolars can depolarize AII amacrines and hyperpolarize OFF cone bipolars, some of which receive rod input. Finally, AII amacrine cells are known to synapse directly onto ganglion cells ^80– 82^, specifically onto certain classes of OFF ganglion cells ^84^. All of these pathways provide opportunities for rod and cone signals to interact.

## Ganglion cells

Recordings from ganglion cells could have special significance for our understanding of rod and cone interactions because ganglion cell responses reflect the aggregate of integration within the whole retina and specify the nature of the signal sent by the retina to the central nervous system. Some ganglion cells receive only cone signals, most notably the midget ganglion cells in the primate fovea, but the large majority appear to receive both rod and cone input from amacrine cells and cone bipolar cells (see, for example, ^53, 85^), allowing their activity to span a large, dynamic range of light intensities.

Although it is possible to stimulate rods and cones selectively with careful selection of light intensity and spectral composition, attempts to investigate rod and cone interactions in ganglion cells have been few and far between (see 8). One of the earliest was the paper of Gouras and Link ^86^, who made extracellular recordings from large (presumably parasol) ganglion cells in the primate perifovea. When brief flashes were given at short intervals to stimulate rods and then cones, or cones and then rods, the first response suppressed the second. Gouras and Link hypothesized that the first response—whether rod or cone—produced a transient inhibition that depressed the second response.

This phenomenon was studied in greater detail and with more powerful techniques nearly 50 years later by Grimes and colleagues ^87^. They also made extracellular recordings from ON parasol cells from primate retina and presented two flashes at short intervals selectively stimulating the rods or cones. They were able to demonstrate clear suppression when stimulation of rods preceded stimulation of cones, but they found little or no effect when the cone signal came first. They then made patch-clamp recordings of voltage responses from horizontal cells, ON cone bipolar cells, and AII amacrine cells, and in each of these cells the rod and cone signals summed linearly. However, they noticed a clear difference in the waveform of rod and cone responses in ON cone bipolar cells: rod responses to brief flashes consisted of a depolarization followed by a pronounced hyperpolarizing undershoot, whereas cone responses were monotonic depolarizations lacking an undershoot. They then constructed a simple model in which rod and cone responses arriving independently at an ON cone bipolar cell were summed linearly and then passed through a common non-linear filter in transit to ganglion cells. This model successfully explained the rod suppression of cone responses, ultimately attributable to the inhibitory undershoot of the rod signal in the cone ON bipolar. Grimes and colleagues showed that this inhibition could be blocked by a cocktail of GABA and glycine receptor inhibitors and may reflect, at least in part, feedback inhibition coming from amacrine cells.

## Conclusions

Although there is a large psychophysical literature describing rod and cone interactions in visual behavior as well as detailed information about the anatomy and physiology of sites within the retina where rod and cone interactions can occur, we still know very little about which sites produce which behaviors. The tools currently available for investigating retinal processing continue to evolve and are vastly more powerful now than even 10 years ago. As these tools improve, we may be able to explain more clearly how the rod and cone systems in the eye function separately and together to produce a coherent perception of the world around us.

## References

[1] Land EH McCann JJ: Lightness and retinex theory. 1971;61(1):1–11. 10.1364/JOSA.61.000001 5541571

[2] Land EH: The retinex theory of color vision. 1977;237(6):108–28. 10.1038/scientificamerican1277-108 929159

[3] Fain GL: Interactions of rod and cone signals in the mudpuppy retina. 1975;252(3):735–69. 10.1113/jphysiol.1975.sp011168 1206574 PMC1348493

[4] McCann JJ Benton JL: Interaction of the long-wave cones and the rods to produce color sensations. 1969;59(1):103–7. 10.1364/JOSA.59.000103 5779636

[5] McCann JJ: Rod-cone interactions: different color sensations from identical stimuli. 1972;176(4040):1255–7. 10.1126/science.176.4040.1255 5033646

[6] Reitner A Sharpe LT Zrenner E: Is colour vision possible with only rods and blue-sensitive cones? 1991;352(6338):798–800. 10.1038/352798a0 1881435

[7] Stabell U Stabell B: Mechanisms of chromatic rod vision in scotopic illumination. 1994;34(8):1019–27. 10.1016/0042-6989(94)90006-X 8160411

[8] Buck SL: Rod-Cone Interactions in Human Vision.In *Visual Neurosciences*, C. LM and W. JS, eds. (Cambridge MA: MIT Press),2003;I:863–878.

[9] MacLeod DI: Rods cancel cones in flicker. 1972;235(5334):173–4. 10.1038/235173a0 4551230

[10] Goldberg SH Frumkes TE Nygaard RW: Inhibitory influence of unstimulated rods in the human retina: evidence provided by examining cone flicker. 1983;221(4606):180–2. 10.1126/science.6857279 6857279

[11] Alexander KR Kelly SA: The influence of cones on rod saturation with flashed backgrounds. 1984;24(5):507–11. 10.1016/0042-6989(84)90048-8 6610987

[12] Mayer C Hafemeister C Bandler RC: Developmental diversification of cortical inhibitory interneurons. 2018;555(7697):457–62. 10.1038/nature25999 29513653 PMC6052457

[13] Nowakowski TJ Bhaduri A Pollen AA: Spatiotemporal gene expression trajectories reveal developmental hierarchies of the human cortex. 2017;358(6368):1318–23. 10.1126/science.aap8809 29217575 PMC5991609

[14] Shekhar K Lapan SW Whitney IE: Comprehensive Classification of Retinal Bipolar Neurons by Single-Cell Transcriptomics. 2016;166(5):1308–1323.e30. 10.1016/j.cell.2016.07.054 27565351 PMC5003425

[15] Helmstaedter M Briggman KL Turaga SC: Connectomic reconstruction of the inner plexiform layer in the mouse retina. 2013;500(7461):168–74. 10.1038/nature12346 23925239

[16] Behrens C Schubert T Haverkamp S: Connectivity map of bipolar cells and photoreceptors in the mouse retina. 2016;5: pii: e20041. 10.7554/eLife.20041 27885985 PMC5148610

[17] Pang JJ Gao F Lem J: Direct rod input to cone BCs and direct cone input to rod BCs challenge the traditional view of mammalian BC circuitry. 2010;107(1):395–400. 10.1073/pnas.0907178107 20018684 PMC2806755

[18] Joesch M Meister M: A neuronal circuit for colour vision based on rod-cone opponency. 2016;532(7598):236–9. 10.1038/nature17158 27049951

[19] Kim IJ Zhang Y Yamagata M: Molecular identification of a retinal cell type that responds to upward motion. 2008;452(7186):478–82. 10.1038/nature06739 18368118

[20] Nath A Schwartz GW: Electrical synapses convey orientation selectivity in the mouse retina. 2017;8(1):2025. 10.1038/s41467-017-01980-9 29229967 PMC5725423

[21] Tikidji-Hamburyan A Reinhard K Storchi R: Rods progressively escape saturation to drive visual responses in daylight conditions. 2017;8(1): 1813. 10.1038/s41467-017-01816-6 29180667 PMC5703729

[22] Aguilar M Stiles WS: Saturation of the Rod Mechanism of the Retina at High Levels of Stimulation. 2010;1(1):59–65. 10.1080/713818657

[23] Makous W: Scotopic Vision. In *Visual Neurosciences* C. LM and W. JS, eds. (Cambridge MA: MIT Press),2003; **I**:838–850.

[24] Naarendorp F Esdaille TM Banden SM: Dark light, rod saturation, and the absolute and incremental sensitivity of mouse cone vision. 2010;30(37):12495–507. 10.1523/JNEUROSCI.2186-10.2010 20844144 PMC3423338

[25] Mendez A Burns ME Sokal I: Role of guanylate cyclase-activating proteins (GCAPs) in setting the flash sensitivity of rod photoreceptors. 2001;98(17):9948–53. 10.1073/pnas.171308998 11493703 PMC55558

[26] Makino CL Dodd RL Chen J: Recoverin regulates light-dependent phosphodiesterase activity in retinal rods. 2004;123(6):729–41. 10.1085/jgp.200308994 15173221 PMC2234569

[27] Chen J Woodruff ML Wang T: Channel modulation and the mechanism of light adaptation in mouse rods. 2010;30(48):16232–40. 10.1523/JNEUROSCI.2868-10.2010 21123569 PMC3010974

[28] Szikra T Trenholm S Drinnenberg A: Rods in daylight act as relay cells for cone-driven horizontal cell-mediated surround inhibition. 2014;17(12):1728–35. 10.1038/nn.3852 25344628

[29] Raviola E Gilula NB: Intramembrane organization of specialized contacts in the outer plexiform layer of the retina. A freeze-fracture study in monkeys and rabbits. 1975;65(1):192–222. 10.1083/jcb.65.1.192 1127010 PMC2111162

[30] Ribelayga C Cao Y Mangel SC: The circadian clock in the retina controls rod-cone coupling. 2008;59(5):790–801. 10.1016/j.neuron.2008.07.017 18786362 PMC5581203

[31] Wu SM Yang XL: Electrical coupling between rods and cones in the tiger salamander retina. 1988;85(1):275–8. 10.1073/pnas.85.1.275 3422423 PMC279527

[32] Asteriti S Gargini C Cangiano L: Mouse rods signal through gap junctions with cones. 2014;3:e01386. 10.7554/eLife.01386 24399457 PMC3882429

[33] Bolte P Herrling R Dorgau B: Expression and Localization of Connexins in the Outer Retina of the Mouse. 2016;58(2):178–92. 10.1007/s12031-015-0654-y 26453550

[34] Hornstein EP Verweij J Li PH: Gap-junctional coupling and absolute sensitivity of photoreceptors in macaque retina. 2005;25(48):11201–9. 10.1523/JNEUROSCI.3416-05.2005 16319320 PMC6725652

[35] Schneeweis DM Schnapf JL: The photovoltage of macaque cone photoreceptors: adaptation, noise, and kinetics. 1999;19(4):1203–16. 10.1523/JNEUROSCI.19-04-01203.1999 9952398 PMC6786037

[36] Deans MR Volgyi B Goodenough DA: Connexin36 is essential for transmission of rod-mediated visual signals in the mammalian retina. 2002;36(4):703–12. 10.1016/S0896-6273(02)01046-2 12441058 PMC2834592

[37] Asteriti S Gargini C Cangiano L: Connexin 36 expression is required for electrical coupling between mouse rods and cones. 2017;34:E006. 10.1017/S0952523817000037 28965521

[38] Lee EJ Han JW Kim HJ: The immunocytochemical localization of connexin 36 at rod and cone gap junctions in the guinea pig retina. 2003;18(11):2925–34. 10.1046/j.1460-9568.2003.03049.x 14656288

[39] O'Brien JJ Chen X Macleish PR: Photoreceptor coupling mediated by connexin36 in the primate retina. 2012;32(13):4675–87. 10.1523/JNEUROSCI.4749-11.2012 22457514 PMC3335500

[40] Jin N Postma F Youn S: The Rod Connexin is Connexin36. 2016;57: Abstract #583.

[41] Kim JW Yang HJ Oel AP: Recruitment of Rod Photoreceptors from Short-Wavelength-Sensitive Cones during the Evolution of Nocturnal Vision in Mammals. 2016;37(6):520–32. 10.1016/j.devcel.2016.05.023 27326930 PMC4918105

[42] Famiglietti EV Jr Kolb H: Structural basis for ON-and OFF-center responses in retinal ganglion cells. 1976;194(4261):193–5. 10.1126/science.959847 959847

[43] Wässle H: Parallel processing in the mammalian retina. 2004;5(10):747–57. 10.1038/nrn1497 15378035

[44] Demb JB Pugh EN: Connexin36 forms synapses essential for night vision. 2002;36(4):551–3. 10.1016/S0896-6273(02)01062-0 12441044

[45] Ingram NT Fain GL Sampath AP: Patch clamp recordings from mouse cone photoreceptores. 2017;58: Abstract #1024.

[46] Euler T Haverkamp S Schubert T: Retinal bipolar cells: elementary building blocks of vision. 2014;15(8):507–19. 10.1038/nrn3783 25158357

[47] Werblin FS Dowling JE: Organization of the retina of the mudpuppy, Necturus maculosus. II. Intracellular recording. 1969;32(3):339–55. 10.1152/jn.1969.32.3.339 4306897

[48] Kaneko A: Physiological and morphological identification of horizontal, bipolar and amacrine cells in goldfish retina. 1970;207(3):623–33. 10.1113/jphysiol.1970.sp009084 5499739 PMC1348731

[49] Ishida AT Stell WK Lightfoot DO: Rod and cone inputs to bipolar cells in goldfish retina. 1980;191(3):315–35. 10.1002/cne.901910302 7410596

[50] Hensley SH Yang XL Wu SM: Relative contribution of rod and cone inputs to bipolar cells and ganglion cells in the tiger salamander retina. 1993;69(6):2086–98. 10.1152/jn.1993.69.6.2086 8350133

[51] Wässle H Yamashita M Greferath U: The rod bipolar cell of the mammalian retina. 1991;7(1–2):99–112. 10.1017/S095252380001097X 1718403

[52] DeVries SH Baylor DA: An alternative pathway for signal flow from rod photoreceptors to ganglion cells in mammalian retina. 1995;92(23):10658–62. 10.1073/pnas.92.23.10658 7479860 PMC40671

[53] Völgyi B Deans MR Paul DL: Convergence and segregation of the multiple rod pathways in mammalian retina. 2004;24(49):11182–92. 10.1523/JNEUROSCI.3096-04.2004 15590935 PMC2834589

[54] Arman AC Sampath AP: Dark-adapted response threshold of OFF ganglion cells is not set by OFF bipolar cells in the mouse retina. 2012;107(10):2649–59. 10.1152/jn.01202.2011 22338022 PMC3362285

[55] Ke JB Wang YV Borghuis BG: Adaptation to background light enables contrast coding at rod bipolar cell synapses. 2014;81(2):388–401. 10.1016/j.neuron.2013.10.054 24373883 PMC4267681

[56] Soucy E Wang Y Nirenberg S: A novel signaling pathway from rod photoreceptors to ganglion cells in mammalian retina. 1998;21(3):481–93. 10.1016/S0896-6273(00)80560-7 9768836

[57] Tsukamoto Y Morigiwa K Ueda M: Microcircuits for night vision in mouse retina. 2001;21(21):8616–23. 10.1523/JNEUROSCI.21-21-08616.2001 11606649 PMC6762784

[58] Mataruga A Kremmer E Müller F: Type 3a and type 3b OFF cone bipolar cells provide for the alternative rod pathway in the mouse retina. 2007;502(6):1123–37. 10.1002/cne.21367 17447251

[59] Tsukamoto Y Omi N: Some OFF bipolar cell types make contact with both rods and cones in macaque and mouse retinas. 2014;8:105. 10.3389/fnana.2014.00105 25309346 PMC4176460

[60] Franke K Berens P Schubert T: Inhibition decorrelates visual feature representations in the inner retina. 2017;542(7642):439–44. 10.1038/nature21394 28178238 PMC5325673

[61] Franke K Baden T: General features of inhibition in the inner retina. 2017;595(16):5507–15. 10.1113/JP273648 28332227 PMC5556161

[62] Kim HG Miller RF: Physiological and morphological correlations of horizontal cells in the mudpuppy retina. 1992;67(4):829–40. 10.1152/jn.1992.67.4.829 1588385

[63] Yang XL Wu SM: Response sensitivity and voltage gain of the rod- and cone-horizontal cell synapses in dark- and light-adapted tiger salamander retina. 1996;76(6):3863–74. 10.1152/jn.1996.76.6.3863 8985884

[64] Kolb H: The connections between horizontal cells and photoreceptors in the retina of the cat: electron microscopy of Golgi preparations. 1974;155(1):1–14. 10.1002/cne.901550102 4836060

[65] Wässle H Boycott BB Peichl L: Receptor contacts of horizontal cells in the retina of the domestic cat. 1978;203(1152):247–67. 10.1098/rspb.1978.0104 84388

[66] Peichl L González-Soriano J: Morphological types of horizontal cell in rodent retinae: a comparison of rat, mouse, gerbil, and guinea pig. 1994;11(3):501–17. 10.1017/S095252380000242X 8038125

[67] Steinberg RH: Rod and cone contributions to *S*-potentials from the cat retina. 1969;9(11):1319–29. 10.1016/0042-6989(69)90069-8 5358837

[68] Trümpler J Dedek K Schubert T: Rod and cone contributions to horizontal cell light responses in the mouse retina. 2008;28(27):6818–25. 10.1523/JNEUROSCI.1564-08.2008 18596157 PMC6670969

[69] Nelson R von Litzow A Kolb H: Horizontal cells in cat retina with independent dendritic systems. 1975;189(4197):137–9. 10.1126/science.1138370 1138370

[70] Baylor DA Fuortes MG O'Bryan PM: Receptive fields of cones in the retina of the turtle. 1971;214(2):265–94. 10.1113/jphysiol.1971.sp009432 5579638 PMC1331836

[71] Babai N Thoreson WB: Horizontal cell feedback regulates calcium currents and intracellular calcium levels in rod photoreceptors of salamander and mouse retina. 2009;587(Pt 10):2353–64. 10.1113/jphysiol.2009.169656 19332495 PMC2697303

[72] Nelson R Kolb H: A17: a broad-field amacrine cell in the rod system of the cat retina. 1985;54(3):592–614. 10.1152/jn.1985.54.3.592 4045539

[73] Chávez AE Grimes WN Diamond JS: Mechanisms underlying lateral GABAergic feedback onto rod bipolar cells in rat retina. 2010;30(6):2330–9. 10.1523/JNEUROSCI.5574-09.2010 20147559 PMC2836865

[74] Hartveit E: Reciprocal synaptic interactions between rod bipolar cells and amacrine cells in the rat retina. 1999;81(6):2923–36. 10.1152/jn.1999.81.6.2923 10368409

[75] Grimes WN Zhang J Graydon CW: Retinal parallel processors: more than 100 independent microcircuits operate within a single interneuron. 2010;65(6):873–85. 10.1016/j.neuron.2010.02.028 20346762 PMC2967021

[76] Dong CJ Hare WA: Temporal modulation of scotopic visual signals by A17 amacrine cells in mammalian retina *in vivo*. 2003;89(4):2159–66. 10.1152/jn.01008.2002 12686583

[77] Kothmann WW Trexler EB Whitaker CM: Nonsynaptic NMDA receptors mediate activity-dependent plasticity of gap junctional coupling in the AII amacrine cell network. 2012;32(20):6747–59. 10.1523/JNEUROSCI.5087-11.2012 22593045 PMC3367513

[78] Mills SL Massey SC: Differential properties of two gap junctional pathways made by AII amacrine cells. 1995;377(6551):734–7. 10.1038/377734a0 7477263

[79] Witkovsky P: Dopamine and retinal function. 2004;108(1):17–40. 10.1023/B:DOOP.0000019487.88486.0a 15104164

[80] Strettoi E Raviola E Dacheux RF: Synaptic connections of the narrow-field, bistratified rod amacrine cell (AII) in the rabbit retina. 1992;325(2):152–68. 10.1002/cne.903250203 1460111

[81] Tsukamoto Y Omi N: Functional allocation of synaptic contacts in microcircuits from rods via rod bipolar to AII amacrine cells in the mouse retina. 2013;521(15):3541–55. 10.1002/cne.23370 23749582 PMC4265793

[82] Tsukamoto Y Omi N: Classification of Mouse Retinal Bipolar Cells: Type-Specific Connectivity with Special Reference to Rod-Driven AII Amacrine Pathways. 2017;11:92. 10.3389/fnana.2017.00092 29114208 PMC5660706

[83] Trexler EB Li W Mills SL: Coupling from AII amacrine cells to ON cone bipolar cells is bidirectional. 2001;437(4):408–22. 10.1002/cne.1292 11503143

[84] Beaudoin DL Kupershtok M Demb JB: Selective synaptic connections in the retinal pathway for night vision. 2017. 10.1002/cne.24313 28856684 PMC5832573

[85] Grimes WN Schwartz GW Rieke F: The synaptic and circuit mechanisms underlying a change in spatial encoding in the retina. 2014;82(2):460–73. 10.1016/j.neuron.2014.02.037 24742466 PMC4038266

[86] Gouras P Link K: Rod and cone interaction in dark-adapted monkey ganglion cells. 1966;184(2):499–510. 10.1113/jphysiol.1966.sp007928 4958644 PMC1357574

[87] Grimes WN Graves LR Summers MT: A simple retinal mechanism contributes to perceptual interactions between rod- and cone-mediated responses in primates. 2015;4: e08033. 10.7554/eLife.08033 26098124 PMC4495655

